# The Motor Impact of the Static Balance in the Up Plank Position on Three Different Balls in Physical Activities of Physical Education Students

**DOI:** 10.3390/ijerph18042043

**Published:** 2021-02-19

**Authors:** Dana Badau, Adela Badau, Gabriel Manolache, Mircea Ion Ene, Adriana Neofit, Vlad Teodor Grosu, Virgil Tudor, Radu Sasu, Raluca Moraru, Liviu Moraru

**Affiliations:** 1 Faculty of Sciences and Letters, “George Emil Palade” University of Medicine, Pharmacy, Sciences and Technology, 540142 Targu Mures, Romania; dana.badau@umfst.ro (D.B.); cristianss4@hotmail.com (R.S.); 2 Faculty of Physical Education and Sport, “Dunarea de Jos” University, 800003 Galati, Romania; gabriel.manolache@ugal.ro (G.M.); mircea.ene@ugal.ro (M.I.E.); adriana.neofit@ugal.ro (A.N.); 3 Faculty of Automotive Mechatronics and Mechanical Engineering, Technical University of Cluj-Napoca, 400114 Cluj-Napoca, Romania; vlad.grosu@mdm.utcluj.ro; 4 Faculty of Physical Education and Sports, National University of Physical Education and Sports, 060057 Bucharest, Romania; virgiltro@yahoo.com; 5 Faculty of Medicine, “George Emil Palade” University of Medicine, Pharmacy, Sciences and Technology, 540142 Targu Mures, Romania; liviu.moraru@umfst.ro

**Keywords:** static balance, plastic balls, up plank position, physical fitness, isometric muscle contraction, health

## Abstract

The present study aimed to assess the capacity of stability and motor impact in the development of the balance of student athletes by reducing the support surface on the ball in the up plank position, by using three categories of balls of equal size, but with different elasticity and weight. In this study, the second aim was to investigate the differences in maintaining static balance, on different balls, between those who are practicing individual sports or team sports. The total study sample include 48 students, active athletes (45.8% of individual sports and 54.2% of team sports), age X ± SD 18.74 ± 1.94 years. The research included two test sessions (initial and final) applied in two stages. The static balance tests were performed by measuring the time maintaining the up plank position with two and three points of support on the three balls, with different characteristics of elasticity and ranges of deformation: medical ball, handball ball and fitness ball. The results of the study showed that the superior initial and final results were recorded on the fitness ball, and the inferior results on the medicine ball. The upper difference was recorded at the up plank position with two support points (arms, legs) on the fitness ball, at 4980 s, and the lowest in the same test on the medical ball, at 3420 s. The largest difference was recorded at the up plank position with three support points on the handball ball, at 7.082 s, and the lowest in the same test on the medical ball, at 3.093 s. The subjects of the study perceived that the most difficult position to execute was the up plank position on the medical ball with two support points, with 43.8%, and the easiest stability was registered in the up plank position on the fitness ball with three points of support, of 37.5%. The relevance of the research results from the possibility of using different balls in conditions of positioning and body posture with a diminished support base in order to improve physical fitness.

## 1. Introduction

In the practice of physical activities, the use of balls of different sizes and with different elastic characteristics is common, and the study of the impact on the components of motor capacity is manifested as a dynamic trend of physical activities, especially in the last decade. The innovative trends of the last decade regarding the design and technologies of different devices and plastic material have largely influenced fields such as medicine, education and sports [1]. Numerous studies have highlighted the fact that the technical characteristics and properties of different sport equipment and materials greatly influence motor capacity in order to optimize physical fitness [2,3].

An important component of physical fitness is balance, which manifests in two forms: statically and dynamically [4,5]. Static balance is an important component and determinant of the body balance and implicit physical fitness. The level of development of body balance is conditioning the development of the other physical skills, which are influenced by the good functionality of the vestibular system [6,7], the central nervous system and the plasticity of the cerebral cortex [8,9,10], the health status [11] and by the level of physical condition and the baggage of individual sports skills [12,13,14]. In practical activity, for the development of body balance, various sports materials are used, with different technical characteristics in terms of shape, elasticity, deformation, dimensions, etc. Studies show that the smaller and more unstable the support surface, the more adequate the motor and postural control must be [15,16,17].

Balls of different compositions, with different characteristics of elasticity and adhesion, as well as with different sizes and weights, are used in the practice of physical activities and in therapy in order to improve the static and dynamic body balance [2,3,4], and the proprioception and strength [18,19,20], as well as in neuro-muscular and functional rehabilitation [21,22,23,24]. The balls that are used in physical activities are made by a specific technology from materials such as butadiene rubber, from polyurethane and polyvinyl chloride. In our study, we used three types of balls, namely: medical balls (butadiene rubber), handball balls (polyurethane) and fitness ball (polyvinyl chloride). Butadiene rubber is an elastic polymer resulting from the polymerization of butadiene, which is the most important monomer and which is characterized by the following properties: good insulator, low elasticity, good water resistance and a remarkable abrasion resistance [25,26]. Polyurethane (PU) balls, a composite material, versatile, stronger and more resistant than PVC, are the closest in terms of technical characteristics to natural leather, with the following properties: high wear resistance, water-repellent effect, softness at low temperatures, and good elasticity allowing for the maintenance of sphericity and good controllability of the ball [27,28]. Polyvinyl chloride (PVC) is a versatile polymer with varied applicability, being a very durable material, with high strength, resistance to climate change and good geometric stability [29]. The research on the effects of the use of different types of balls on the parameters of static balance and general force focused on the development of throwing force [30] and improvement in technical sports skills [31,32].

Investigating the up plank position under various conditions of balance and strength, using fitness equipment with specific dimensions and technical characteristics, facilitates the understanding of motor impact by facilitating the extension of its applicability in physical activities and therapy [33,34,35,36]. The up plank position is a complex movement that requires maintaining balance in conditions of isometric muscle contraction. Anatomical analysis of maintaining an up plank position highlights the activation of the following muscles: biceps brachii, triceps brachii, deltoideus, brachialis, latissimus dorsi, serratus anterior, obliquus internus, vastus lateralis, rectus femoris and tibialis anterior, as well as articulations: scapulo-humeral, humero-radio-ulnar, radio-carpal, inter-carpal, coxo-femural, femuro-tibial, tibio-talar and inter-tarsal [37,38]. The up plank position is used in fitness activities in order to improve body balance and general muscle strength, aiming at the development and rehabilitation of the vestibular system and the human muscular system. The up plank position is also used as a fitness test to assess muscle strength and endurance [39,40,41,42]. In our study, we want to give a new use to this plank up test in order to evaluate the ability to maintain static balance on small and unstable surfaces.

The study focused on highlighting the capacity of stability and motor impact in the development of balance and implicit of the general strength by reducing the support surface on the ball in the up plank position, by using three categories of balls of equal size, but of different elasticity and weight. It is important to understand how the use of different fitness equipment, with different technical characteristics, influences the components of physical fitness in physical activities.

In this context, the first aim of the study was to evaluate the stable equilibrium capacity in the top board position with two and three support points in relation to the characteristics and technical properties of the use of three types of ball with the same shape and circumference, with different elasticities and weights, namely: medical ball, handball and fitness ball. Because the research sample consisted of active athletes customized according to the type of sport, the second objective of the study was to investigate the differences between those who practice individual sports and those who practice team sports aiming to maintain static balance on the three balls selected for the study.

## 2. Materials and Methods

### 2.1. Participants

The study included a sample of volunteer students from the University of Medicine, Pharmacy, Science and Technology “George Emil Palade” of Targu Mures, Romania. All participants were informed about the details of the study; all participants were anonymous and voluntary. The inclusion criteria in the study were: active student in the Physical Education and Sports program, active athlete, good health, age under 20. We selected only male students because the number of female students in the Physical Education and Sports program is very small, ≤10, in accordance with the inclusion criteria. The sample included 48 male students, 22 students of individual sports (45.8%), 26 students of team sports (54.2%), with a mean age X ± SD 18.74 ± 1.94 years; mean height X ± SD 178.39 ± 6.60 cm; average weight X ± SD 75.35 ± 6.56 kg.

### 2.2. Procedure

The study took place between 28 September and 29 November 2020. The study was conducted in accordance with the Declaration of Helsinki, and the protocol was approved by the the Review Board of Movement Sciences Department, “George Emil Palade” University of Medicine, Pharmacy, Sciences and Technology of Targu Mures, Romania (resolution no. 36/23/05/2019). The research included two test sessions applied in two stages: initial testing and final testing. The initial testing was applied at the beginning of the experiment between 28 September 28 and 30 September 2020, and the final testing between 27 and 29 November 2020.

### 2.3. Program of the Static Balance

Between the initial and final tests, the independent variable consisting of a program provided online including balance exercises and the development of general muscle strength was applied, three times per week, with 20 min per session. The balance exercise program included free exercises with a diminished support surface and stable balance exercises on different balls with different sizes and elasticities: medicine balls, handball balls, fitness balls, boss ball, swiss fitness ball. The study participants were monitored online via the google meets platform; the exercise program was performed at home. Before each training session, participants received the daily descriptive and imaging program including a description of the exercises, the dosage, the nature and duration of the break between the exercise evenings, and the sports materials used. The training sessions, as well as the monitoring of the correctness of the execution, were carried out under the verbal and video guidance of the study experts.

### 2.4. Measures

We selected the plank up fitness test on unstable round surfaces for this study. The plank up test has been used in numerous studies to measure global core muscle function, with a focus on balance capacity, endurance and muscle strength [40,41,42]. The static balance tests were performed by measuring the time spent maintaining the up plank position with two and three points of support on the three balls: the medical rubber ball, the synthetic leather handball ball and the PVC fitness ball. The timer is turned on (measuring the time in seconds) from the moment when the subject is in the correct position, maintaining balance, and it is stopped when the subject is unbalanced and is touching the ground. For this study, we used three different kind of balls, with variable characteristics of elasticity. Ball materials used in the study are as follows: ballasted medical balls made of 100.00% butadiene rubber, weight 1 kg, diameter 19 cm, recommended pressure 0.3–0.5 bar; handball balls Select size 3 made of polyurethane (ultragrippy PU synthetic leather, sewn by hand), with latex chamber, weight 0.475 kg, diameter 19 cm, recommended pressure 0.3–0.5 bar; fitness ball made PVC, weight 0.340 kg, diameter 19 cm, recommended pressure 0.3–0.5 bar.

### 2.5. Research Instruments

The tests took place in the gym under the direct organization and evaluation of the experts from the study. The tests had the following chronology: session 1—testing the static balance in the up plank position on the medical ball with two support points (arms—legs), then a 30 min break followed by testing the static balance in the up plank position on the handball ball with two support points (arms—legs), then a 30 min break followed by testing the static balance in the up plank position on the fitness ball with two support points (arms—legs); session 2—held 2 days after the first session, as follows: testing the static balance in the up plank position on the medicine ball with three support points (right arm—left arm—legs), then a 30 min break followed by testing the static balance in the up plank position on handball ball with three points of support (left arm—right arm—legs), then a 30 min break followed by testing the static balance in the up plank position on fitness ball with three points of support (left arm—right arm—legs).

### 2.6. Data Analysis

Statistical analysis of the plank up test results for evaluating the static balance on round, elastic and unstable surfaces were processed with the program SPSS 21., calculating the following basical statistical indicators: arithmetic mean (X), standard deviation (SD), Min.—Minimum, Max.—Maximum, XD—differences between arithmetic averages, SDD—differences between standard deviations. The normality of distributions was assessed by using the Shapiro–Wilk test (S-W). Differences between groups were analysed with Student’s *t*-test. To detect the true effect, we calculated the statistical power (SP) for the repeated measures and the chosen level required was at least 0.8. Selected significance level of probability was *p* ≤ 0.05.

## 3. Results

The analysis of the results in Table 1 from the motor evaluation shows that between the balance tests on the three balls with 2 and 3 support points, the superior initial and final results were recorded on the fitness ball, and the inferior results on the medicine ball. The comparative analysis of the results shows a statistically significant power; the registered values were higher than the threshold of 0.80. All results were statistically significant for *p* ≤ 0.05. All the data were normally distributed; the null hypothesis is rejected.

**Figure 1:**
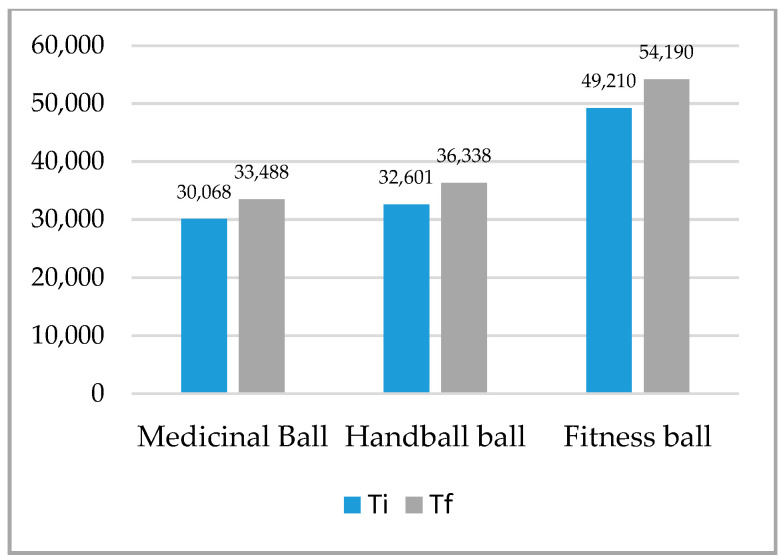
Mean of plank up test with 2 support points (arms, legs). Ti—initial test; Tf—final test.

**Figure 2:**
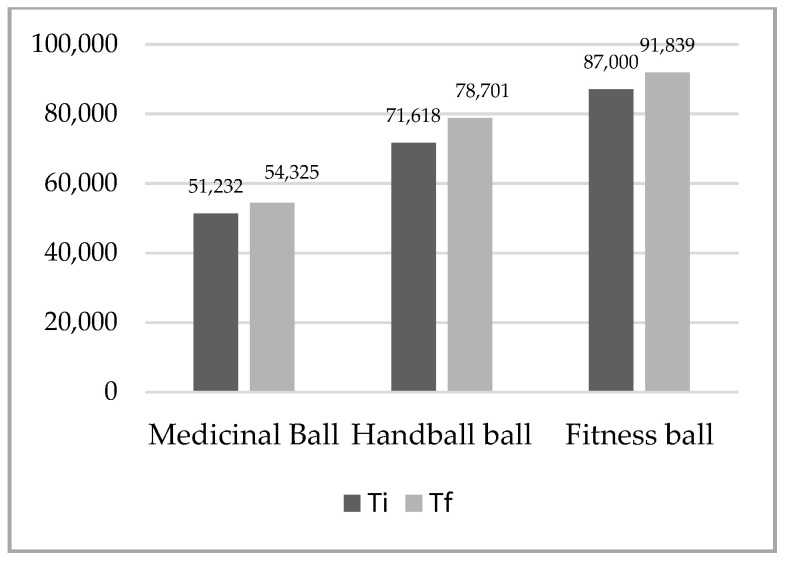
Mean of plank up test with 3 support points (arms, legs).

The difference analyses between initial and final tests for maintaining the balance test on the three types of balls in the up plank position for the whole sample were statistically significant for both tests and on all three balls (Table 2, Figure 1, Figure 2). The higher difference was recorded at the up plank position with two support points (arms, legs) on the fitness ball DX 4.980, and the lowest at the same test on the medical ball DX 3.420. The largest difference was recorded at the up plank position with three support points (right arm, left arm, legs) on the handball ball DX 7.082, and the lowest at the same test on the medical ball DX 3.093.

**Figure 3:**
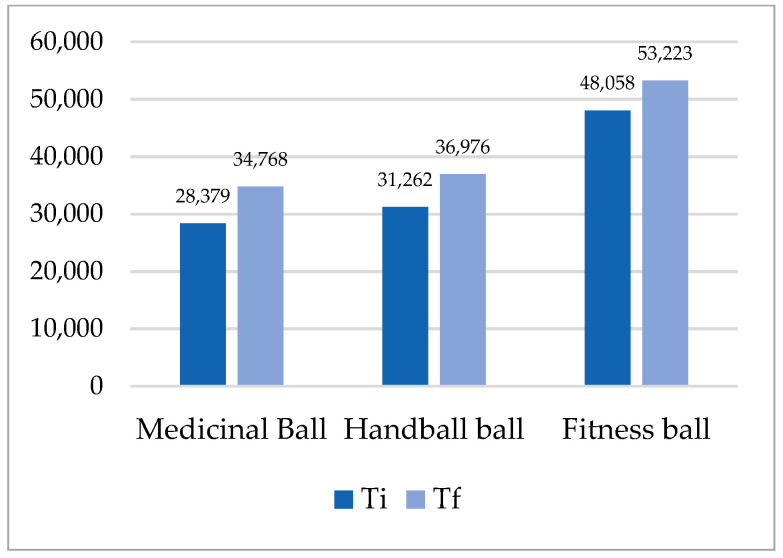
Mean of plank up test with 2 support points (arms, legs)—individual sports.

**Figure 4:**
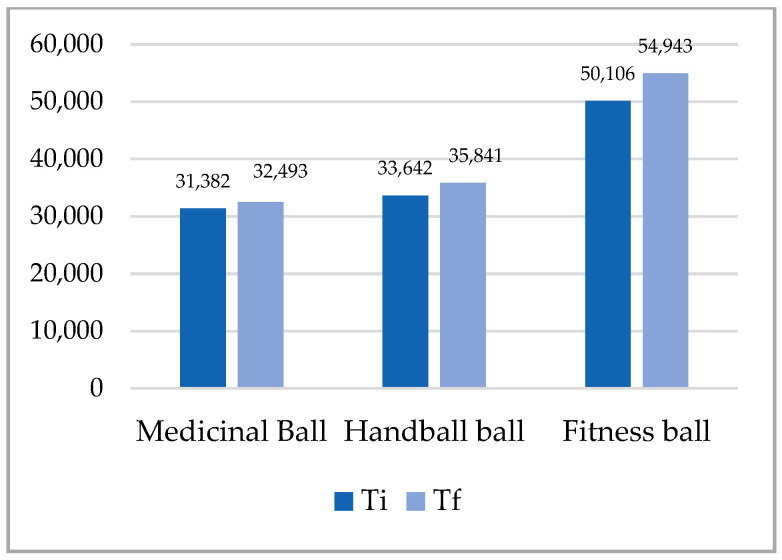
Mean of plank up test with 2 support points (arms, legs)—team sports.

**Figure 5:**
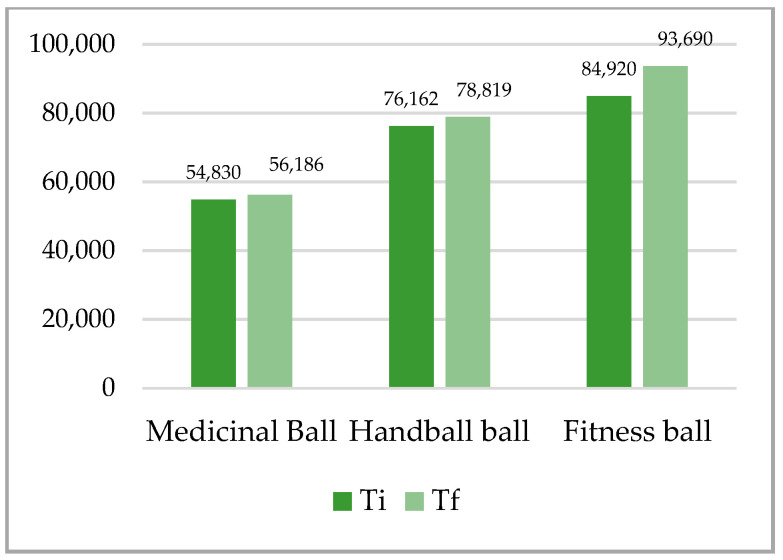
Mean of plank up test with 3 support points (arms, legs)—individual sports.

**Figure 6:**
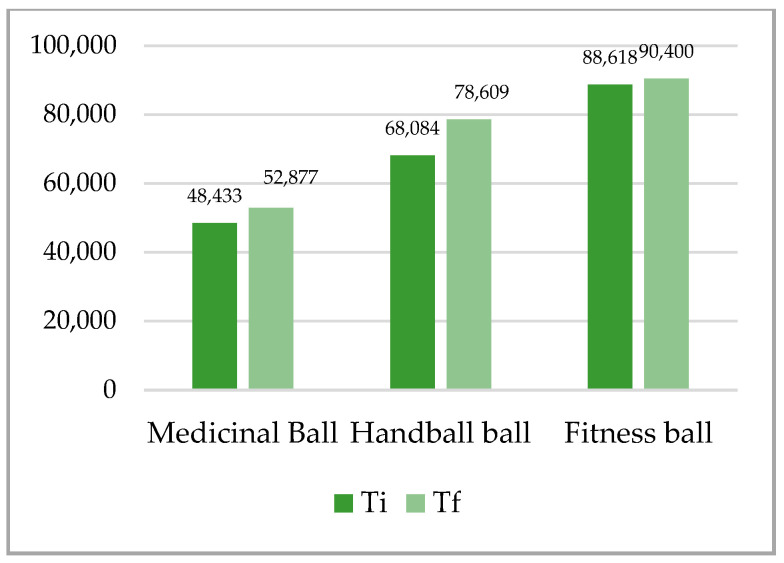
Mean of plank up test with 3 support points (arms, legs)—team sports.

The analysis of the results in Table 3, Figure 3, Figure 4, Figure 5 and Figure 6, shows that the superior results were recorded in both tests on the fitness ball, and the inferior results were shown on the medicine ball. In the individual sports group, the highest value was recorded in the final test in the up plank position test with three support points (right arm, left arm, legs) on the fitness ball 93.690, and the lowest in the initial test in the up test plank position with three support points (right arm, left arm, legs) on the fitness ball 28.379. The analysis of the team sports group shows that the highest value was recorded at the final test in the up plank position test with three support points (right arm, left arm, legs) on the fitness ball 90.400, and the lowest at the initial test at the test up plank position with three support points (right arm, left arm, legs) on the fitness ball 31.382. The distribution of the results was normal, according to the S-W results, which ranged between 0.795 and 0.923. All results in both initial and final tests for both groups were statistically significant.

The difference analyses between initial and final tests for maintaining balance show that the smallest differences were recorded for both groups on the medical balls, and the best results were recorded for the group of individual sport on the fitness ball DX 8.770, and for group of team sport on handball DX 10.524. The comparative analysis of the results shows a significant statistical power; the recorded values were higher than the threshold of 0.80. The results were not statistically significant, with one exception for the up plank position with three support points (right arm, left arm, legs) on the handball ball for the team sports group (Table 4).

The results in Table 5, which analyzes the average differences recorded at the initial test between the individual sports group and group sports team, at both tests, show statistically insignificant differences, with one exception for the up plank position test with three support points (right arm, left arm, legs) on the medical ball. The comparative analysis of the results shows a significant statistical power; the recorded values were higher than the threshold of 0.80, between 0.807 and 0.861.

## 4. Discussion

The first aim of the study was to identify the differences regarding the static balance capacity in the up plank position on three different types of balls in terms of elasticity and degree of deformation. The results show significant differences in the static balance test in the up plank position on the three balls, in favor of the fitness ball, due to the superior elasticity and the higher degree of deformation compared to handball and medicinal balls. The spherical shape of the balls combine with the elasticity and the deformation capacity, determines the complex motor adaptation the subjects must perform in order to maintain their balance. The results of the study reflect that the shape and structure of the support surface significantly influences the static equilibrium capacity. The differences between the final and the initial testing reveal that the implementation of some balance exercise programs by using sports materials made of elastic materials and with spherical shapes were effective. The results of the study complement previous studies that have shown that maintaining the up plank position on different balls required the multimodal integration of sensory information, combined with postural and vestibular control on unstable surfaces [43,44,45].

The second aim was to investigate the differences in maintaining static balance on different balls between those who are practicing individual sports or team sports. The results of the study highlight the better static balance ability of subjects who practice team sports compared to those who practice individual sports. Highlighting the differences between the two categories of athletes in the study reflects the impact of differences in motor experience, the complexity of physical training, and the complexity of technical skills specific to team sports compared to the particularities of sports training in individual sports.

The results of the study are in agreement with the results of previous studies [12,18,46,47], with the particularity that they aim to use the up plank position as an exercise to develop balance; in our study, this position was used both as an exercise and as a motor assessment test. Some studies have focused on studying the impact of the shape and technical characteristics of sports materials on physical performance [46,47], with a focus on the efficiency of motor and technical capacities [48,49]. The specialists in prophylaxis and physiotherapy focused on adapting the use of materials in the process of motor recovery and functional rehabilitation, depending on the technical characteristics and the individual and pathological particularities of the subjects [50,51,52]. The trend of using different sports and fitness materials is becoming increasingly obvious, and their area of use is increasingly extensive, including fitness centers and sport activities. The technical and compositional characteristics of these materials used can have major influences on physical capacity, perception of effort, body posture and vestibular and neuromotor rehabilitation. Consistent with the results of our study, previous studies have highlighted the influence of exercises performed on different fitness materials in order to improve static and dynamic balance [5,53,54,55]. The results of our study complete the research on the study of the up plank position, which were numerous and especially highlighted the anatomical, biomechanical and physical benefits of different categories of subjects actively involved in physical and sports activities [56,57,58,59], in the practice of fitness [55,60,61,62,63] and in prophylaxis [64,65,66,67,68].

Our study focused on evaluating the duration of maintaining the up plank position in conditions of static balance reduced to two and three points of support on balls that have spherical shapes, different characteristics of elasticity, and varying degrees of deformity. The results of our study were statistically significant in the case of evaluating the entire sample. Regarding the comparison of the individual sports group with the team sports group, it was highlighted that those from team sports have superior indices of balance and superior strength, revealed by the period of maintenance of balance on duration of all the tests and on all three variants of balls. The results of the study contribute to the understanding of the effects on human physical capacity and of the exercise modalities determined by the technical characteristics of sports materials in physical activities and those of prophylactic activities.

The practical implications of the study will be focused on the use of balls of various sizes and with different technical characteristics in order to improve the static balance. We recommend the use of balls with a high elasticity characteristic, especially in the first phase of exercise or prophylaxis, then continue with balls with lower elastic characteristics and low degrees of deformation that require increased fitness skills. Exercises on balls to improve static balance are recommended for all categories of athletes, mainly for practitioners of complex sports that involve balance skills, such as gymnastics, aerobics and fitness.

The main strengths of this study are the relatively large number of participants who met the inclusion criteria, and the number of tests performed in the up plank position with the reduction in the support surface to two and three points on three balls with the same circumference, but with different elastic characteristics, different deformation capacities and different weights. The results may be relevant in both the practice of physical activities in order to improve motor control, postural control and to improve static balance and general muscle strength. The limits of the identification research are the period of study was limited because of changes in the educational system from partial onside into total online, non-identification of the degree of muscular tension in performing the tests for highlighting the most activated muscle groups when maintaining the up plank position, and the non-involvement of a sample of student girls, due to the small number of total students in the academic program targeted in the research.

## 5. Conclusions

The results of the study confirm that a higher elasticity of the balls positively influences the results regarding the physical performances, aiming at a static balance in the up plank position. The results of the balance test on the fitness ball with two and three support points were superior to those recorded in the tests on handball and medical balls. The superior results registered at the tests on the handball and fitness balls are considered to be determined by the superior characteristics of elasticity and, implicitly, its deformation, in comparison with the medical balls that have a reduced elasticity. The results recorded by the study subjects reflect the fact that, on the medical ball, the perception of balance is better than on the handball and fitness balls, which are more elastic and easier, and therefore have greater possibilities of deformation, and the joint and muscle demand is higher. The study shows that students playing team games showed a greater capacity for static balance on spherical and elastic surfaces than those who practice individual sports. These results could have practical connotations regarding the extension of exercise programs to improve balance by using fitness materials which are as varied as possible and with characteristics of elasticity, shape, hardness, etc., that are as diverse as possible. The results of athletes playing team games compared to those in individual sports can be correlated with the greater complexity of technical training, which can have a positive influence on the level of body balance. The relevance of the research results from the possibility of using different balls in conditions of positioning and body posture with a diminished support base in order to improve physical fitness focused on functional, vestibular and neuromuscular prophylaxy and rehabilitations.

## Figures and Tables

**Table 1:** Differences between initial and final tests of physical tests of maintaining balance on the three types of ball in the up plank position—for whole sample.

Positions	Types of Balls	Tests	X (s)	SD	Min. (s)	Max (s)	S-W
Up plank position with 2 support points (arms, legs)	on the medical ball	Ti	30.068	11.233	8.62	56.12	0.817
		Tf	33.488	15.840	10.62	91.71	0.893
	on the handball ball	Ti	32.601	16.957	9.27	77.45	0.855
		Tf	36.338	21.678	10.62	85.32	0.891
	on the fitness ball	Ti	49.210	26.737	15.11	98.27	0.907
		Tf	54.190	33.207	18.58	120.27	0.875
Up plank position with 3 support points (right arm, left arm, legs)	on the medical ball	Ti	51.232	12.429	31.12	75.13	0.907
		Tf	54.325	13.485	34.59	84.59	0.893
	on the handball ball	Ti	71.618	30.499	28.44	132.12	0.869
		Tf	78.701	35.024	34.65	148.60	0.875
	on the fitness ball	Ti	87.000	35.303	34.78	156.13	0.808
		Tf	91.839	40.778	48.92	174.25	0.821

X—Mean, SD—Std. Deviation, Min.—Minimum, Max.—Maximum, S-W—Shapiro–Wilk test, Ti—initial test, Tf—final test.

**Table 2:** Descriptive statistics of difference analyses between initial and final tests for maintaining balance test on the three types of balls in the up plank position—for whole sample.

Positions	Types of Balls	Tests	X (sec.)	DX (sec.)	DSD	t	p	SP
Up plank position with 2 support points (arms, legs)	on the medical ball	Ti	30.068	−3.420	10.963	−2.161	0.036	0.826
		Tf	33.488					
	on the handball ball	Ti	32.601	−3.737	11.537	−2.244	0.030	0.815
		Tf	36.338					
	on the fitness ball	Ti	49.210	−4.980	16.411	−2.103	0.041	0.897
		Tf	54.190					
Up plank position with 3 support points (right arm, left arm, legs)	on the medical ball	Ti	51.232	−3.093	9.6511	−2.220	0.031	0.882
		Tf	54.325					
	on the handball ball	Ti	71.618	−7.082	19.811	−2.477	0.017	0.873
		Tf	78.701					
	on the fitness ball	Ti	87.000	−4.839	14.737	−2.275	0.028	0.849
		Tf	91.839					

X–Mean, DX—differences of mean, DSD—differences of Std. Deviation, *t*-value of Student test, SP—statistical power, p—level of probability, Ti—initial test, Tf—final test.

**Table 3:** Differences between initial and final tests of physical tests of maintaining balance on the three types of balls in the up plank position—individual sports group and team sports group.

Groups	Positions	Types of Balls	Tests	X (s)	SD	Min. (s)	Max (s)	S-W
Individual sports (22 n)	Up plank position with 2 support points (arms, legs)	on the medical ball	Ti	28.379	10.844	8.62	41.63	0.897
			Tf	34.768	19.569	10.62	91.71	0.892
		on the handball ball	Ti	31.262	12.360	10.62	50.87	0.866
			Tf	36.976	21.496	10.62	85.32	0.872
		on the fitness ball	Ti	48.058	24.745	15.11	91.71	0.903
			Tf	53.223	32.488	18.58	120.27	0.923
	Up plank position with 3 support points (right arm, left arm, legs)	on the medical ball	Ti	54.830	12.726	31.12	75.13	0.893
			Tf	56.186	12.632	34.59	75.13	0.914
		on the handball ball	Ti	76.162	30.098	34.65	118.60	0.795
			Tf	78.819	38.531	34.65	148.60	0.807
		on the fitness ball	Ti	84.920	33.577	34.78	125.13	0.893
			Tf	93.690	41.973	48.92	174.25	0.869
Team sports (26 n)	Up plank position with 2 support points (arms, legs)	on the medical ball	Ti	31.382	11.555	10.62	56.12	0.875
			Tf	32.493	12.517	10.62	56.97	0.808
		on the handball ball	Ti	33.642	19.992	9.27	77.45	0.821
			Tf	35.841	22.214	10.62	85.32	0.817
		on the fitness ball	Ti	50.106	28.623	18.58	98.27	0.866
			Tf	54.943	34.353	18.58	120.27	0.872
	Up plank position with 3 support points (right arm, left arm, legs)	on the medical ball	Ti	48.433	11.666	32.88	70.20	0.819
			Tf	52.877	14.178	34.65	84.59	0.816
		on the handball ball	Ti	68.084	30.903	28.44	132.12	0.871
			Tf	78.609	32.793	34.65	132.02	0.869
		on the fitness ball	Ti	88.618	37.141	48.92	156.13	0.875
			Tf	90.400	40.569	48.92	174.25	0.866

X—Mean, SD—Std. Deviation, Min.—Minimum, Max.—Maximum, S-W—Shapiro–Wilk test, Ti—initial test, Tf—final test, n—number of subjects.

**Table 4:** Descriptive statistics of difference analyses between initial and final tests for maintaining balance test on the three types of ball in the up plank position—individual sports group and team sports group.

Groups	Positions	Types of Balls	Tests	X (s)	DX (s)	DSD	t	*p*	SP
Individual sports (22 n)	Up plank position with 2 support points (arms, legs)	on the medical ball	Ti	28.379	−6.389	14.921	−1.962	0.064	0.876
			Tf	34.768					
		on the handball ball	Ti	31.262	−5.714	14.687	−1.783	0.090	0.902
			Tf	36.976					
		on the fitness ball	Ti	48.058	−5.165	15.774	−1.501	0.149	0.858
			Tf	53.223					
	Up plank position with 3 support points (right arm, left arm, legs)	on the medical ball	Ti	54.830	−1.355	5.470	−1.136	0.269	0.802
			Tf	56.186					
		on the handball ball	Ti	76.162	−2.657	18.122	−0.672	0.509	0.815
			Tf	78.819					
		on the fitness ball	Ti	84.920	−8.770	20.602	−1.951	0.065	0.862
			Tf	93.690					
Team sports (26 n)	Up plank position with 2 support points (arms, legs)	on the medical ball	Ti	31.382	−1.111	5.773	−1.000	0.327	0.807
			Tf	32.493					
		on the handball ball	Ti	33.642	−2.199	8.311	−1.375	0.181	0.832
			Tf	35.841					
		on the fitness ball	Ti	50.106	−4.836	17.187	−1.462	0.156	0.848
			Tf	54.943					
	Up plank position with 3 support points (right arm, left arm, legs)	on the medical ball	Ti	48.433	−4.444	11.875	−1.945	0.063	0.836
			Tf	52.877					
		on the handball ball	Ti	68.084	−10.524	20.705	−2.641	0.014	0.829
			Tf	78.609					
		on the fitness ball	Ti	88.618	−1.782	6.629	−1.397	0.174	0.842
			Tf	90.400					

X—mean, DX—differences of mean, DSD—differences in Std. Deviation, t—value of Student test, SP—statistical power, p—level of probability, Ti—initial test, Tf—final test, n—number of subjects.

**Table 5:** Descriptive statistics for physical tests of maintaining balance on the three types of balls in the up plank position.

Positions	Types of Balls	Tests	X (s)	SD	t	*p*	SP
Up plank position with 2 support points (arms, legs)	on the medical ball	Ti: individual vs. team sports	−3.003	17.479	−0.621	0.542	0.823
		Tf: individual vs. team sports	2.591	22.033	0.539	0.596	0.861
	on the handball ball	Ti: individual vs. team sports	−1.102	24.522	-0.206	0.839	0.826
		Tf: individual vs. team sports	1.783	32.325	0.253	0.803	0.849
	on the fitness ball	Ti: individual vs. team sports	1.174	41.342	0.130	0.898	0.828
		Tf: individual vs. team sports	0.121	53.633	0.010	0.992	0.841
Up plank position with 3 support points (right arm, left arm, legs)	on the medical ball	Ti: individual vs. team sports	5.982	12.946	2.118	0.047	0.847
		Tf: individual vs. team sports	1.624	17.709	0.420	0.679	0.818
	on the handball ball	Ti: individual vs. team sports	7.611	42.180	0.827	0.418	0.852
		Tf: individual vs. team sports	4.271	47.728	0.410	0.686	0.819
	on the fitness ball	Ti: individual vs. team sports	1.147	49.289	0.107	0.916	0.807
		Tf: individual vs. team sports	8.488	53.216	0.731	0.473	0.817

X–Mean, SD—Std. Deviation, t—value of Student test, SP—statistical power, Ti—initial test, Tf—final test, vs.—versus.

## Data Availability

Not applicable.
